# Rapid and Ultrasensitive Detection of *Staphylococcus aureus* by a One-Pot System Integrating *Pyrococcus furiosus* Argonaute with Loop-Mediated Isothermal Amplification

**DOI:** 10.4014/jmb.2504.04034

**Published:** 2025-07-14

**Authors:** Guangda Li, Jiajun Wang, Lei Tian, Mingchao Ding, Yang Liu, Jingfu Wang

**Affiliations:** 1 Department of Stomatology, General Hospital of Northern Theater Command, 83 Wenhua Road, Shenyang, P.R. China; 2 Stomatology College of Jiamusi University, Jiamusi, P.R. China; 3 State Key Laboratory of Oral & Maxillofacial Reconstruction and Regeneration, National Clinical Research Center for Oral Diseases, Shaanxi Clinical Research Center for Oral Diseases, Department of Oral and Maxillofacial Surgery, School of Stomatology, The Fourth Military Medical University, Xi’an, P.R. China; 4 Department of Otolaryngology, Head and Neck Surgery, The 901th Hospital of the Joint Logistics Support Force of the Chinese People’s Liberation Army, Hefei, P.R. China; 5 The First Hospital of Qiqihar, Qiqihar, P.R. China

**Keywords:** *Staphylococcus aureus*, *Pyrococcus furiosus* Argonaute, loop-mediated isothermal amplification (LAMP), nucleic acid detection

## Abstract

*Staphylococcus aureus* (*S. aureus*) is one of the most common pathogens associated with oral and maxillofacial space infections (OMSI), significantly impairing patients' quality of life and posing substantial public health risks. Traditional detection methods are usually time-consuming and operationally complex, which limits their applicability for rapid on-site detection. This study introduces a novel, ultrasensitive nucleic acid detection system combining loop-mediated isothermal amplification (LAMP) with *Pyrococcus furiosus* Argonaute (*Pf*Ago) in a one-pot strategy. The combination of LAMP and *Pf*Ago not only improves specificity and sensitivity but also effectively reduces the risk of aerosol contamination and false positives. The system detects the *nuc* gene of *S. aureus*, enabling rapid detection within 50 min with a limit of detection (LOD) of 10^0^ Colony Forming Units (CFU)/ml for bacterial solutions and 10^-5^ ng/μl for plasmids. This system exhibited exceptional specificity by effectively differentiating *S. aureus* from other common OSMI bacteria, and was successfully applied to samples from animal OMSI infection models. This innovative one-pot system offers a rapid, reliable, and effective solution for *S. aureus* detection, with potential applications in human health and public safety.

## Introduction

The oral and maxillofacial region, situated at the onset of the digestive and respiratory tracts, naturally harbors a substantial microbial population. When the equilibrium between microorganisms and the host is disturbed, infections can arise [1]. Oral and maxillofacial space infection (OMSI) is a common clinical condition, often causing symptoms such as local swelling, pain, and limited mouth opening, which seriously reduces the quality of life of patients [2]. The primary cause of OMSI is odontogenic factor. In addition to odontogenic origins, OMSI may also result from cysts, salivary gland inflammation, facial carbuncles, furuncles, and other conditions [3]. The loose tissue structure of the oral and maxillofacial spaces, combined with interconnections between these spaces, enables rapid disease spread, causing extensive multi-space infections over a short period. It exerts a substantial influence on both facial morphology and function, as well as mental health conditions. If not effectively controlled, the infection may disseminate to other regions of the body, potentially causing cavernous sinus thrombophlebitis, brain abscesses, mediastinitis, sepsis, ulcerative endocarditis, osteomyelitis, nephritis, and severe complications such as asphyxia, which can endanger the patient's life [4-7]. *Staphylococcus aureus* is one of the most common pathogens associated with OMSI and is also among the most prevalent pathogens globally [8, 9]. Each year, millions of cases of *Staphylococcus aureus* infections are reported, ranging from mild skin infections to life-threatening conditions such as pneumonia, endocarditis, and sepsis [10]. Consequently, the development of rapid and effective detection methods for *S. aureus* is critical for the prevention, diagnosis, and treatment of OMSI.

The traditional methods for bacterial detection include bacterial culture and others [11]. The bacterial culture method is considered the “gold standard” for bacterial detection due to its high accuracy and comprehensive ability to detect bacterial drug resistance [12-15]. It remains widely adopted in numerous hospitals to this day. However, it has notable limitations. For instance, the detection process typically requires at least 2-3 days, with some cases extending up to a week, which fails to meet the urgent diagnostic and clinical medication needs of patients with acute infections. Additionally, during the prolonged culture period, dominant bacteria may overgrow and suppress less dominant ones, altering the composition and proportion of pathogenic bacteria responsible for the infection and thereby affecting the final detection results [14, 16]. To address these limitations, molecular biology-based detection methods have been developed, including enzyme-linked immunosorbent assay (ELISA), polymerase chain reaction (PCR), and loop-mediated isothermal amplification (LAMP) and others [17-19]. Among these, PCR is currently regarded as the “gold standard” in molecular biology-based detection methods [20]. These techniques have significantly reduced detection time and improved the efficiency of clinical diagnosis. Nevertheless, they also have certain drawbacks, such as complex operational requirements, and a tendency to produce cross-reactions. For example, PCR necessitates a professional laboratory, precise detection equipment, and skilled technical personnel. In recent years, rapid nucleic acid detection technology based on *Pyrococcus furiosus* Argonaute (*Pf*Ago) has been developed and has shown rapid advancements [21-23].

In 2004, the Argonaute protein in *P. furiosus* was first identified [23]. As a nucleic acid-guided endonuclease, *Pf*Ago recognizes, guides, and cleaves target genes using 5'-phosphorylated guide DNA (gDNA). The technical principle involves designing two gDNA segments based on the target gene sequence. The gDNA binds to *Pf*Ago, activating its enzymatic activity and enabling cleavage of the target gene sequence at the position between the 10th and 11th nucleotides at the 5' end of the gDNA sequence. Fluorescent probes are designed based on the single-stranded DNA (ssDNA) fragments generated by cleavage. These ssDNA fragments can also serve as secondary gDNA, recombining with *Pf*Ago to cleave the fluorescent probes. When cutting the fluorescence probe, the black hole quencher (BHQ) group moves away from the fluorescein amidite (FAM) group, and the fluorescence probe emits specific fluorescence. *Pf*Ago is widely used in various fields such as pathogen identification and genetic disease screening due to its excellent performance, simple operation, and fast, accurate detection effect, making it an important tool in modern biotechnology [24, 25]. The advent of *Pf*Ago-based detection technology has provided novel research approaches and methodologies for the rapid, high-precision, and efficient detection of *S. aureus* [22]. In 2019, He *et al*. introduced a nucleic acid detection method leveraging *Pf*Ago, termed *Pf*Ago-mediated Nucleic Acid Detection (PAND). This was the first approach to exploit the unique characteristics of *Pf*Ago for pathogen detection [26]. Li *et al*. reported a novel biosensor based on *Pf*Ago (NOTE-Ago) for detecting *Salmonella typhi* and *S. aureus*, achieving a limit of detection (LOD) of 1 CFU/ml [15]. Chen *et al*. developed a nucleic acid detection system combining *Pf*Ago and RPA technology for the diagnosis of respiratory tract *S. aureus* infections. This system completes detection within 55 min with an LOD of 10^2^ copies/μl [27]. Kou *et al*. devised an Argonaute-centered portable and visual biosensor for the detection of clinical samples, contaminated foods, and *MRSA*-infected animals, achieving an LOD of 10 CFU/ml within 65 min [28]. This study expands the scope of Argonaute-based biosensing applications and introduces a groundbreaking bacterial point-of-need (PON) detection platform.

Detection methods based on *Pf*Ago often need to be combined with nucleic acid amplification technology, and the development of a one-pot detection strategy is currently the main research direction. This is because one-pot detection sensors offer advantages such as ease of operation, reduced risk of contamination, and fast, efficient performance. Due to these advantages, one-pot detection schemes have been increasingly applied in point-of-care testing (POCT). Li *et al*. developed a one-pot detection system that integrates LAMP and CRISPR/Cas12b technology for SARS-CoV-2 detection, achieving a LOD as low as 10 copies/reaction. This method demonstrates significant advantages in simplifying procedures, reducing costs, and enhancing detection efficiency [29]. Han *et al*. developed a one-tube SARS-CoV-2 detection platform based on RTX-PCR and *Pyrococcus furiosus* Argonaute. The LOD was 100 copies/ml, and the diagnostic results of clinical samples showed 100% consistency with RT-qPCR testing[30]. The one-pot strategy has the advantages of miniaturization and portability, and also achieves the goal of not opening the tube cover during the entire reaction process, which will not cause aerosol contamination and minimize false positive.

Herein, a One-pot *Pf*Ago detection system (Opps) was developed, which achieves fast and sensitive detection of *S. aureus*. This system achieves rapid detection of *S. aureus* within 50 min, with a LOD of 10^0^ CFU/ml for bacterial solutions and 10^-5^ ng/μl for plasmids. At the same time, this study also achieved precise detection of animal samples infected by *S. aureus*. Therefore, this system not only significantly shortens the detection time and simplifies the experimental operation process but also effectively minimizes the risk of cross-contamination. The successful application of this system in detecting *S. aureus* demonstrates its potential for adaptation to other pathogens, establishing a versatile platform that can be rapidly tailored to address emerging infectious diseases. This advancement positions our system as a critical component in future pandemic preparedness and response strategies.

## Materials and Methods

### Reagents and Materials

*S. aureus* ATCC 25923 was provided by The Fourth Military Medical University (China). The conserved sequences corresponding to the *nuc* (GenBank: OQ992662.1) genes of *S. aureus* was retrieved from the National Center for Biotechnology Information (NCBI) database. The *nuc* gene, encoding the thermostable nuclease specific to *S. aureus*, was selected as the detection target due to its high specificity and conservation within *S. aureus* strains, and it an ideal biomarker to ensure minimal cross-reactivity. All LAMP and *Pf*Ago reagents, as well as all oligonucleotides and fluorescent probe (Table S1) used in this experiment were provided and synthesized by JiaoHong Biotech (China).

### Design and Screening of LAMP Primers, gDNA and Probe

For the LAMP assay, three sets of primers were specifically designed using Primer Premier 5.0 software (Eiken Chemicals Co., Japan) based on the conserved region of the *nuc* gene of *S. aureus*. The probe was designed as a ssDNA sequence, incorporating a FAM fluorescent label at the 5' end and a black hole quencher (BHQ) at the 3' end. LAMP primers were screened by comprehensive analysis of the fluorescence intensity. Under the action of LAMP primers, a ssDNA was amplified as the target gene sequence. Three complementary 16 bp gDNAs with 5'-phosphorylation were designed based on the amplified sequence for gDNA screening. Select the gDNA combination with the highest fluorescence intensity of the fluorescent cleavage reaction.

### Optimization of Opps

To obtain the optimal conditions for the detection, this experiment conducted optimization experiments on two key factors in the reaction: reaction temperature and *Pf*Ago mass. The overall reaction temperature conditions were systematically optimized by testing a range of temperatures (86°C, 89°C, 92°C, 95°C, 98°C) to identify the most suitable condition for the reaction using 10^7^ CFU/ml bacterial solution. Under the optimal reaction temperature conditions, the effects of different *Pf*Ago mass were evaluated (76 μg, 152 μg, 228 μg, 304 μg). Quantitative assessment of fluorescence intensity, combined with a detailed analysis of reaction efficiency parameters, facilitated the determination of the optimal *Pf*Ago mass.

### Sensitivity and Specificity of Opps

In the one-pot system, the following components were added to each *Pf*Ago reaction assay with a total volume of 25 μl. The 25 μl *Pf*Ago reaction contained 1.0 μl Bst 4.2 DNA polymerase, 2.5 μl Mg^2+^ (40 mM), 1.5 μl Buffer solution, 3.5 μl dNTP, 1.25 μl LAMP amplification primer for *nuc* gene, 1.0 μl 5'-phosphorylated gDNA (20 μM), 1 μl fluorescent probe (10 μM), 1.0 μl pyrophosphatase, 7.75 μl double distilled water (ddH2O), 5.0 μl DNA template and *Pf*Ago endonuclease. The reaction mixture was amplified at a constant temperature of 70°C for 20 min, and then heated to 95°C for *Pf*Ago cleavage reaction.

In the one-pot system, the aforementioned method was utilized to perform sensitivity experiments on both *S. aureus* plasmid and bacterial solution. The prepared standard was diluted 10-fold serially using sterile enzyme-free water to obtain plasmid concentrations ranging from 10^-1^ to 10^-5^ ng/μl and bacterial solution concentrations ranging from 10^1^ to 10^7^ CFU/ml for sensitivity detection. Specificity experiments of the reaction systems were carried out for the five common pathogens associated with OMSI using 10^7^ CFU/ml bacterial solution, including *S. aureus*, *Streptococcus mutans*, *Bacteroides fragilis*, *Klebsiella pneumoniae* and *Granulicatella adiacens*. All tests were independently repeated thrice.

### Establishment of OMSI Infection Animal Models

To assess the applicability of Opps in real animal infection samples. The study was conducted in compliance with the guidelines for the ethical review of laboratory animal welfare as outlined in the People’s Republic of China National Standard (GB/T 35892-2018) and was approved by the Institutional Animal Care and Use Committee of the Fourth Military Medical University (Approval No. kq-2024-150). Twelve male Sprague-Dawley rats were randomly selected and randomly divided into two groups, which were then numbered. The Sprague-Dawley rats underwent a 3-day adaptive feeding period. To expose the skin, the hair on the maxillofacial region of the experimental Sprague-Dawley rats was removed using a combination of depilatory cream and a razor. The control group received daily submandibular space injections of 200 μl of 0.9% normal saline, whereas the infection group received daily submandibular space injections of 200 μl of 10^8^ CFU/ml bacterial solution. Submandibular region swelling was monitored in both groups over a 72-h period.

### Statistics and Analysis

All the figures in this article were generated using GraphPad Prism software. Data were analyzed with GraphPad Prism version 10.1.2 software (GraphPad Software Inc., USA) using one-way analysis of variance (ANOVA). These values were significantly different compared to the control group. N = 3 technical replicates, *P < 0.05, **P < 0.01, ***P < 0.001, ****P < 0.0001.

## Results and Discussion

### LAMP Primers and gDNA Screening

**Fig. 1:**
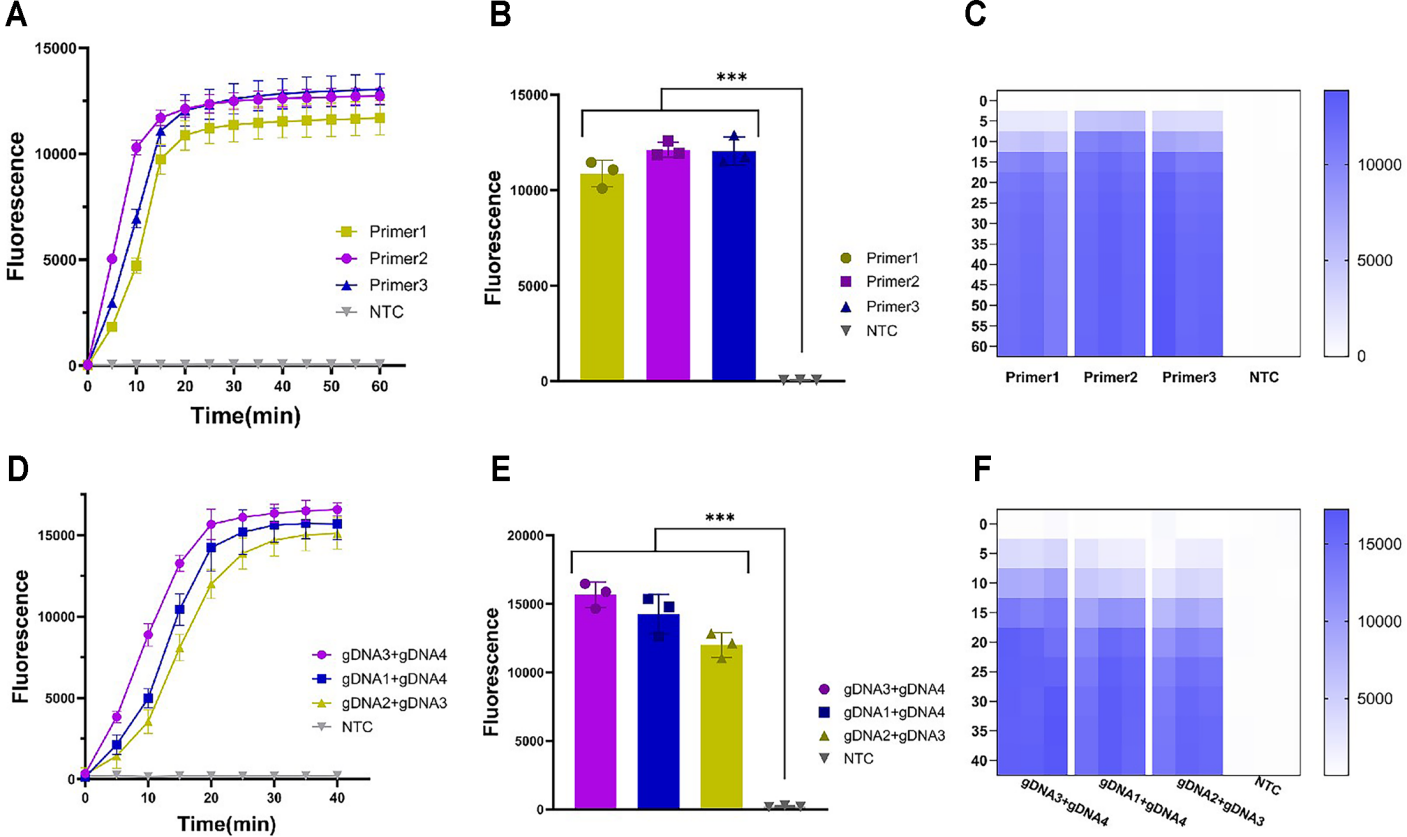
LAMP Primers and gDNA Screening. (**A**) Fluorescence curve for the screening of LAMP primers. (**B**) 20-min endpoint fluorescence histogram of one-pot system LAMP primers screening. (**C**) Heat map of LAMP primers screening. (**D**) Fluorescence curve for the screening of gDNA. (**E**) 20-min endpoint fluorescence histogram of one-pot system gDNA screening. (**F**) Heat map of gDNA screening. Three replicates were run (*n* = 3). NTC, non-template control reaction. Error bars represent the means ± standard deviation (s.d.) from replicates.

This study performed a systematic primers screening experiment to evaluate the efficiency of amplification primers used in LAMP assays. Three primer combinations were carefully designed for the specific *nuc* gene fragment of *S. aureus*. Through fluorescence intensity analysis, the Primer2 generated the strongest fluorescence signal, exhibited the earliest peak onset, and demonstrated the highest amplification efficiency within 20 min (Fig. 1A-1C). Therefore, the Primer2 is considered the optimal LAMP primer for subsequent one-pot system detection.

Next, this study conducted a screening analysis of gDNA, a critical factor in the *Pf*Ago cleavage reaction. The combination of gDNA2 and gDNA3, gDNA1 and gDNA4 and the combination of gDNA3 and gDNA4 were designed to determine the optimal set (Table S1). Through fluorescence intensity analysis, the combination of gDNA3 and gDNA4 generated the highest fluorescence signal compared to all other combinations. Within a 20-min time frame, this combination consistently demonstrated the highest fluorescence signal intensity at any given time, indicating satisfactory reaction efficiency (Fig. 1D-1F). Therefore, the combination of gDNA3 and gDNA4 was chosen as the guiding DNA for the one-pot system detection.

The LAMP amplification method exhibits satisfactory stability in comparison to other amplification techniques. Due to the excellent anti-interference ability of LAMP [31], the detection reaction can still be completed even when mixed with various gene sequences and interference. LAMP primers are a critical component in amplification reactions. Well-designed primers can ensure highly efficient amplification, thereby facilitating subsequent detection reactions. Each set of LAMP primers comprises a pair of outer primers (F3 and B3), a pair of inner primers (FIP and BIP), as well as LF and LB primers. From a theoretical perspective compared to PCR detection, which requires only two primers, LAMP employs a greater number of primers, thereby achieving higher specificity and more accurate amplification reactions [32]. Simultaneously, another key element of one-pot system is gDNA, which can guide the cleavage reaction of *Pf*Ago. An optimal combination of gDNA can enhance reaction performance, thereby improving the efficiency of the cleavage reaction and increasing the sensitivity and specificity of detection [33-35]. Thus, the selection of LAMP primers and gDNA plays a critical role in ensuring the efficiency and reliability of the one-pot system detection reaction. Therefore, the Primer3 is considered the optimal LAMP primer for subsequent one-pot system detection, and the combination of gDNA3 and gDNA4 was chosen as the guiding DNA for the one-pot system detection.

### Optimization of the Opps

**Fig. 2:**
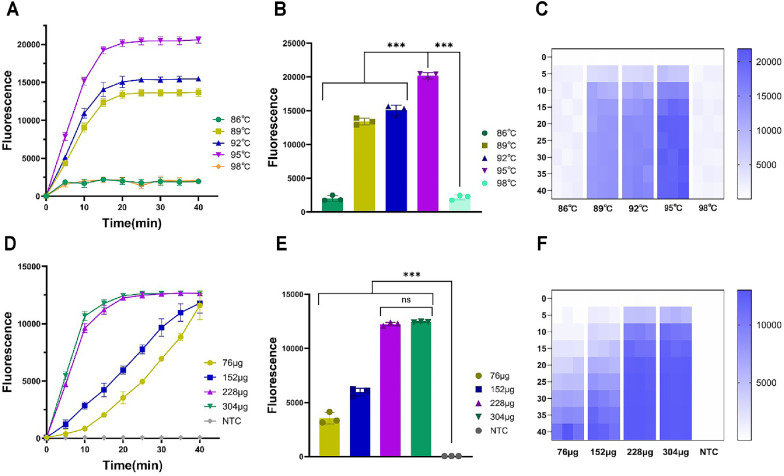
Optimization of the Opps. (**A**) Fluorescence curve for the optimization of reaction temperature. (**B**) 20-min endpoint fluorescence histogram of optimization of reaction temperature. (**C**) Heat map of optimization of reaction temperature. (**D**) Fluorescence curve for the screening of *Pf*Ago mass optimization. (**E**) 20-min endpoint fluorescence histogram of *Pf*Ago mass optimization. (**F**) Heat map of *Pf*Ago mass optimization. Three replicates were run (*n* = 3). NTC, non-template control reaction. Error bars represent the means ± standard deviation (s.d.) from replicates.

The reaction temperature and the quality of *Pf*Ago are two crucial factors in the Opps, directly influencing the reaction efficiency and the detection results. Robust quantification of fluorescence signals and evaluation of reaction kinetics parameters facilitated precise identification of the optimal reaction temperature and *Pf*Ago mass, thereby establishing the most efficient reaction system configuration. This study carried out optimization under different reaction temperature conditions to determine the optimal reaction temperature for the Opps, thereby ensuring maximal reaction efficiency. The overall reaction temperature conditions were systematically optimized by testing a range of temperatures (86°C, 89°C, 92°C, 95°C, 98°C) to identify the most suitable condition for the reaction. As shown in Figure 2A-2C, the fluorescence intensity reaches its maximum at a reaction temperature of 95°C. Moreover, within 20 min, a reaction temperature of 95°C exhibits a good fluorescence reaction efficiency. Accurate temperature control is critical for ensuring the smooth progression of the reaction. If the temperature is too low, it may fail to initiate the *Pf*Ago-mediated cleavage reaction or result in incomplete cleavage. Conversely, excessively highs can lead to the loss of endonuclease activity [36, 37]. Thus, precise and stable temperature regulation is essential for maintaining the efficiency of the reaction system.

Under the optimal reaction temperature conditions, further condition optimization experiments were performed to evaluate the effects of different *Pf*Ago mass (76 μg, 152 μg, 228 μg, 304 μg) on the reaction efficiency. The experiment found that when the mass of *Pf*Ago was 228 μg, the fluorescence reaction would reach its maximum value, and good experimental results would be obtained. Moreover, when the mass of *Pf*Ago increased from 228 μg to 304 μg, it was found that there was no significant change in the experimental results (Fig. 2D-2F). Therefore, the reaction conditions were optimized to a temperature of 95°C and a *Pf*Ago mass of 228 μg. The mass of *Pf*Ago directly influences both reaction efficiency and experimental outcomes. An insufficient mass of *Pf*Ago can lead to incomplete reactions within the reaction system, thereby compromising detection results. Conversely, an excessively high mass of *Pf*Ago not only fails to enhance reaction efficiency further but may also suppress it, ultimately affecting the accuracy of detection results [38, 39]. Therefore, the mass of *Pf*Ago play a critical role in ensuring the efficiency and stability of the reaction system.

### Sensitivity and Specificity of Opps

**Fig. 3:**
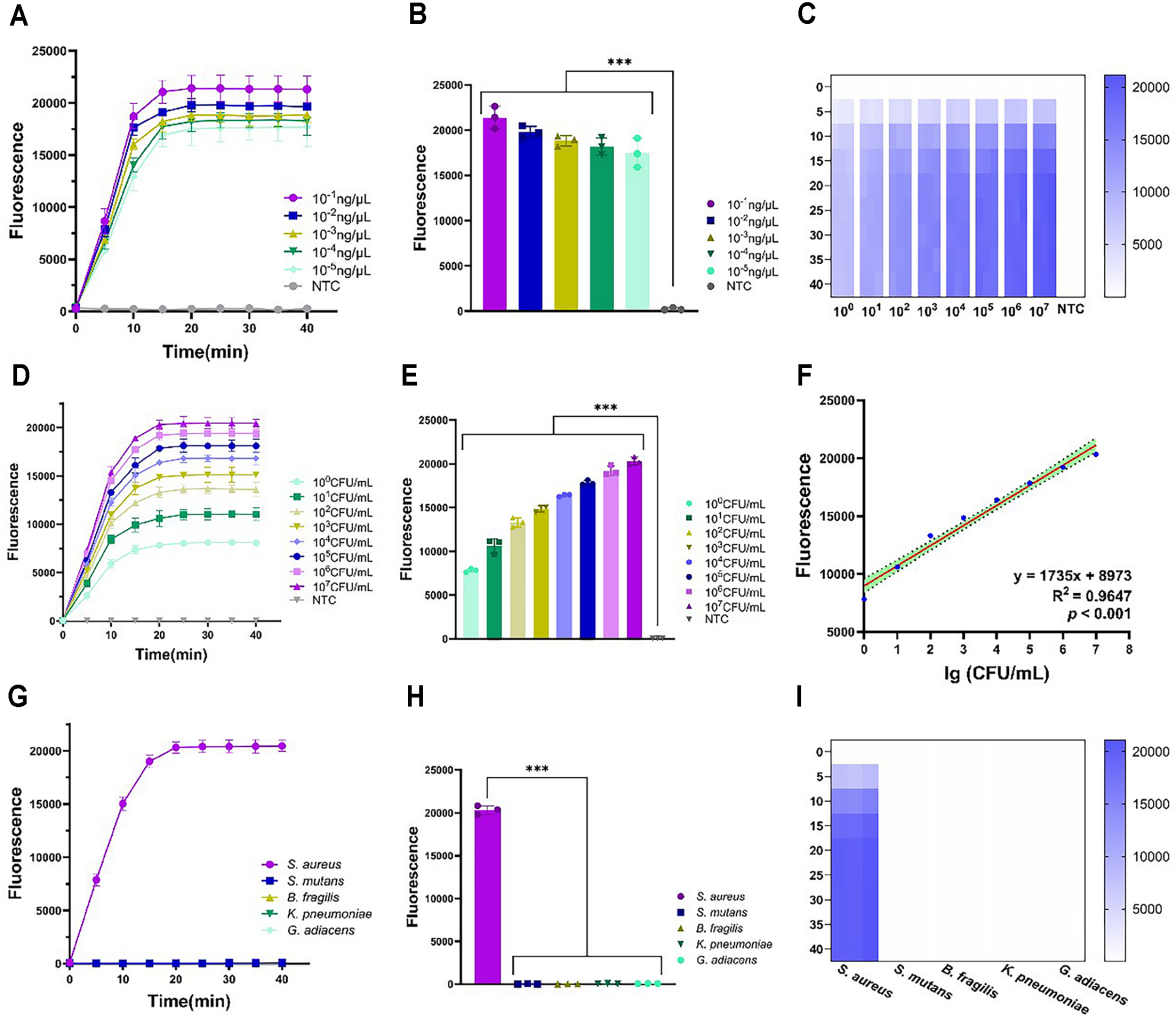
Sensitivity and Specificity of Opps. (**A**) Fluorescence curve of plasmid sensitivity detection. (**B**) 20-min endpoint fluorescence histogram of plasmid sensitivity detection. (**C**) Heat map of plasmid sensitivity detection. (**D**) Fluorescence curve of sensitivity detection of *S. aureus* solution. (**E**) 20-min endpoint fluorescence histogram of sensitivity detection of *S. aureus* solution. (**F**) (**I**) Linear relationship of serial-diluted concentration of *S. aureus* solution and fluorescence intensity. (**G**) Fluorescence curve of specificity detection. (**H**) 20-min endpoint fluorescence histogram of specificity detection. (**I**) Heat map of specificity detection. Three replicates were run (*n* = 3). NTC, non-template control reaction. Error bars represent the means ± standard deviation (s.d.) from replicates.

To comprehensively assess the detection performance of Opps, this study conducted experiments focusing on sensitivity and specificity. 10-fold diluted plasmid ranging from 10^-1^ to 10^-5^ ng/μl and bacterial solutions ranging from 10^0^ to 10^7^ CFU/ml were detected by the system. As illustrated in Figure 3A and 3B, the fluorescence intensity of the bacterial plasmid was significantly higher than that of the negative control group, indicating a clear distinction between the positive and control groups across all tested concentrations. Given that satisfactory detection results were achieved at all these concentrations, the lowest concentration of 10^-5^ ng/μl was selected as the LOD. Meanwhile, the bacterial solution exhibited detection effect same to that observed in plasmid. The significant difference in fluorescence intensity, when compared to the negative control group, clearly demonstrated the distinction between the positive samples and the controls. Additionally, all tested concentrations yielded ideal detection outcomes, the LOD reach 10^0^ CFU/ml (Fig. 3C-3E). The Opps achieves a LOD of 10^0^ CFU/ml for bacterial solutions, surpassing traditional culture methods and rivaling even advanced PCR-based assays. Notably, this sensitivity is achieved within 50 min, a stark contrast to the 24–72 h required for culture-based identification [40]. Compared to other *Pf*Ago-based platforms, Opps demonstrates comparable or superior performance[24, 41]. For instance, Chen *et al*. achieved 10^2^ copies/μl using RPA-*Pf*Ago in 55 min [27]. The Opps shorter detection time (50 min) and ability to detect both plasmids and bacterial solutions without separate processing steps highlight its efficiency. Additionally, the one-pot design minimizes cross-contamination risks.

The ideal testing outcome would be characterized by high accuracy, specificity, and reproducibility, reflecting the optimal performance of the detection method. Figure 3F illustrates the relationship between the logarithm of bacterial solution concentration and fluorescence intensity using a linear regression equation. The linear regression equation is y = 1735x + 8973 (R² = 0.9647). As the concentration of the bacterial solution increases, the fluorescence intensity exhibits a corresponding increase, demonstrating a strong linear correlation between the two variables. In addition, five common pathogens associated with OMSI were carried out to evaluate the specificity of the Opps. As shown in Figure 3G-3I, fluorescence signals only appear when *S. aureus* is present, indicating the excellent specificity of the Opps. The *nuc* gene is widely recognized as a conserved and species-specific marker for *S. aureus* detection. To address sequence variability, we analyzed publicly available genomic data of NCBI for *nuc* sequences across major *S. aureus* lineages, such as ST72 and USA300. It was found that the *nuc* gene (GenBank: OQ992662.1) fragment amplified in this study could be found with completely identical gene sequences in both the ST72 lineage and USA300 fragments. Thus, in this study, the *nuc* gene was chosen as the target for detection due to its high specificity and conservation across *S. aureus* strains.

Therefore, the LOD of the system achieved 10^-5^ng/μl in plasmid and 10^0^ CFU/ml in bacterial solution, and this system exhibits excellent specificity. Compared with other *Pf*Ago-based detection methods, Opps demonstrates same specificity [42, 43], suggesting its potential as a highly effective detection system.

### Establishment of OMSI Infection Animal Models

**Fig. 4:**
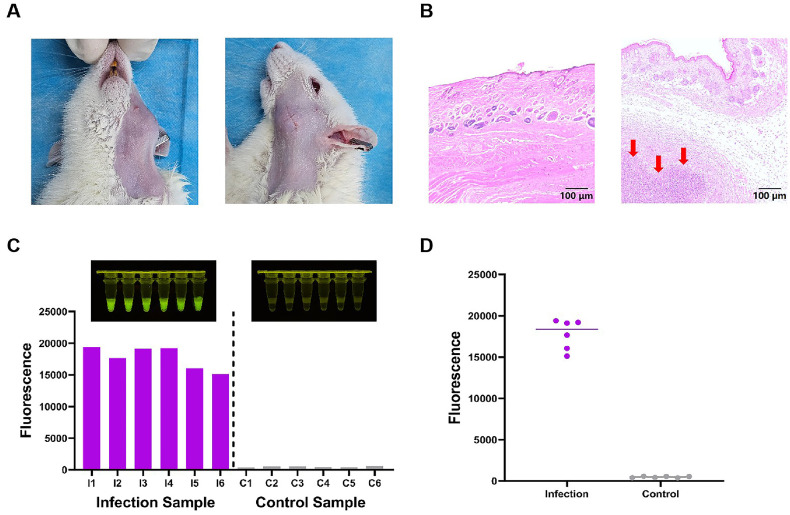
Establishment of OMSI Infection Animal Models. (**A**) Images of the rat infection model. (**B**) Images of HE staining in soft tissue (Red arrows indicate areas of inflammatory cell infiltration). (**C**) Sensing signal response of infection and control rat samples. (**D**) Significant difference analysis of the signals of rat samples.

To assess the applicability of Opps in real animal infection samples. First, establish an infection-based animal model. The establishment of OMSI infection animal model was validated based on observations of swelling in the submandibular space, pus formation, and histopathological confirmation through the hematoxylin and eosin (HE) staining images (Fig. 4A and 4B). Subsequently, 12 test samples were collected from each of the 12 Sprague-Dawley rats (6 Sprague-Dawley rats in the infection group and 6 Sprague-Dawley rats in the control group) and tested individually using Opps. The test results are presented in Figure 4C and 4D. A significant fluorescence difference was observed between the infection group and the control group, demonstrating that the system achieved 100% accuracy in sample detection. The detection capability of this system in animal samples is comparable to that of other methods based on *Pf*Ago [44], thereby validating the good detection performance of Opps in infected animal models.

The successful application of Opps in animal OMSI models underscores its translational relevance. The 100% concordance between infected and control samples suggests robustness in complex biological matrices. Furthermore, the system operational simplicity positions it as a viable tool for POCT. This aligns with global trends toward decentralized diagnostics, particularly in low-resource settings where OMSI burden is high but laboratory infrastructure is limited. Future iterations could integrate Opps with portable, battery-operated devices, as demonstrated in recent *Pf*Ago-based visual biosensors, to enable real-time, on-site diagnosis.

The integration of LAMP and *Pf*Ago leverages the unique strengths of both technologies. LAMP eliminates the need for thermal cycling, enabling rapid nucleic acid amplification even in resource-limited settings. However, LAMP alone is prone to non-specific amplification and aerosol contamination, which can compromise specificity. The incorporation of *Pf*Ago addresses these limitations through its DNA-guided endonuclease activity. Unlike CRISPR-based systems that rely on protospacer adjacent motifs (PAM sequences) and require additional trans-cleavage reporters, *Pf*Ago directly cleaves target DNA at specific sites guided by phosphorylated gDNA. This dual-step mechanism—amplification followed by sequence-specific cleavage—ensures that only true targets generate detectable fluorescence signals, effectively minimizing false positives. Furthermore, the use of two gDNAs (gDNA3 and gDNA4) in Opps, enhancing specificity by recognition of regions within the *nuc* gene. This design principle aligns with recent advances in multiplexed *Pf*Ago systems, where multi-guide strategies improve discrimination between closely related bacterial species. While Opps exhibits remarkable performance, several challenges merit consideration. First, the requirement for 95°C during the *Pf*Ago cleavage phase may limit compatibility with heat-labile components in field settings. Although *Pf*Ago is thermostable, prolonged high temperatures could degrade fluorescent probes or other reagents. Second, a critical next step is to evaluate Opps against a broad cohort of human clinical samples, stratified by infection severity, comorbidities, and prior antibiotic exposure, to assess its robustness in real-world scenarios. This would strengthen the translational relevance of the system and address potential gaps in diagnostic accuracy when deployed in heterogeneous patient populations.

## Conclusion

The One-pot *Pf*Ago detection system successfully established a rapid and highly sensitive method for detecting *S. aureus* in OMSI patients, namely Opps. The detection technology of this system employs specific LAMP primers as well as the specific recognition and precise cleavage capabilities of *Pf*Ago. The proposed system demonstrates excellent sensitivity and enables rapid detection within 50 min with a LOD of 10^0^ CFU/ml for bacterial solution and 10^-5^ ng/μl for plasmid. Moreover, the system exhibits high specificity, with no cross-reactivity to non-*S. aureus* strains. This high sensitivity and specificity render the system highly promising for practical sample applications. Meanwhile, Opps demonstrated satisfactory detection performance in samples obtained from animal infection models. It achieves a faster detection time compared to traditional methods, and the results also exhibit high repeatability. Overall, the development of this study holds great significance for the detection of *S. aureus*. The Opps provides a rapid, sensitive, and reliable method for detecting *S. aureus*. At the same time, it provides robust support for the prevention and control of *S. aureus* infections in OMSI and enhances the capacity to safeguard public health and hygiene safety.

## Supplemental Materials

Supplementary data for this paper are available on-line only at http://jmb.or.kr.
