# Supplementary material for: Rapid and Ultrasensitive Detection of Staphylococcus aureus by a One-Pot System Integrating Pyrococcus furiosus Argonaute with Loop-Mediated Isothermal Amplification
Source: J Microbiol Biotechnol. 2025 Jul 14;35:e2504034. doi: 10.4014/jmb.2504.04034 (PMC12283255; doi:10.4014/jmb.2504.04034)
Supplement: Supplementary file 1 [file jmb-35-e2504034-supple.pdf]

J. Microbiol. Biotechnol. 2025. 35: e254034

https://doi.org/10.4014/jmb.2504.04034

Supplementary Table

Rapid and Ultrasensitive Detection of *Staphylococcus aureus* by a One-Pot System Integrating *Pyrococcus furiosus* Argonaute with Loop-Mediated Isothermal Amplification

Guangda Li[2,3], Jiajun Wang[5], Lei Tian[3], Mingchao Ding[3*], Yang Liu[4*], and Jingfu Wang[1*]

[1]Department of Stomatology, General Hospital of Northern Theater Command, 83 Wenhua Road, Shenyang, P.R. China [2]Stomatology College of Jiamusi University, Jiamusi, P.R. China [3]State Key Laboratory of Oral & Maxillofacial Reconstruction and Regeneration, National Clinical Research Center for Oral Diseases, Shaanxi Clinical Research Center for Oral Diseases, Department of Oral and Maxillofacial Surgery, School of Stomatology, The Fourth Military Medical University, Xi'an, P.R. China [4]Department of Otolaryngology, Head and Neck Surgery, The 901th Hospital of the Joint Logistics Support Force of the Chinese People's Liberation Army, Hefei, P.R. China [5]The First Hospital of Qiqihar, Qiqihar, P.R. China

Table S1. Oligonucleotides primers and gDNA.

Oligonucleotides		Sequence
Primer 1	F3	AACAGTATATAGTGCAACTTCAA
	B3	CTTTGTCAAACTCGACTTCAA
	FIP	TGTCATTGGTTGACCTTTGTACATTTTACATAAAGAACCTGCGACA
	BIP	GATACACCTGAAACAAAGCATCCTATTTTTTTCGTAAATGCACTTGC
	LF	CCGTATCACCATCAATCGCTTTA
	LB	AAAGGTGTAGAGAAATATGGCCCTG
Primer 2	F3	AAAAATTACATAAAGAACCTGCG
	B3	CAGTTCTTTGACCTTTGTCA
	FIP	TGTCATTGGTTGACCTTTGTACAACATTAATTAAAGCGATTGATGGTG
	BIP	AAGGTGTAGAGAAATATGGCCCTCTCGACTTCAATTTTATTTGCA
	LB	GAAGCAAGTGCATTTACGAA
Primer 3	F3	TTGTAGTTTCAAGTCTAAGTAGC
	B3	CACCTTTTTAGGATGCTTTG
	FIP	ATACTGTTGGATCTTCAGAACCACTCAGCAAATGCATCACAAACA
	BIP	AAGCGATTGATGGTGATACGGTAGGTGTATCAACTAATAATAGTCTG
	LB	AATGTACAAAGGTCAACCAATGACA
gDNA 1		TTAGTTGATACACCTG
gDNA 2		TAACAAAGCATCCTAA
gDNA 3		TAGTTGATACACCTGA
gDNA 4		TACAAAGCATCCTAAA
Probe		TGATACACCTGAAACAAAGCATCC
